# Targeting glioma stem‐like cell survival and chemoresistance through inhibition of lysine‐specific histone demethylase KDM2B

**DOI:** 10.1002/1878-0261.12174

**Published:** 2018-02-12

**Authors:** Mikkel Staberg, Rikke Darling Rasmussen, Signe Regner Michaelsen, Henriette Pedersen, Kamilla Ellermann Jensen, Mette Villingshøj, Jane Skjoth‐Rasmussen, Jannick Brennum, Kristoffer Vitting‐Seerup, Hans Skovgaard Poulsen, Petra Hamerlik

**Affiliations:** ^1^ Department of Radiation Biology The Finsen Center Copenhagen University Hospital Denmark; ^2^ Brain Tumor Biology Group Danish Cancer Society Research Center Copenhagen Denmark; ^3^ Department of Neurosurgery Copenhagen University Hospital Denmark

**Keywords:** cancer stem‐like cell, chemoresistance, epigenetics, glioblastoma, histone demethylase

## Abstract

Glioblastoma (GBM) ranks among the most lethal cancers, with current therapies offering only palliation. Inter‐ and intrapatient heterogeneity is a hallmark of GBM, with epigenetically distinct cancer stem‐like cells (CSCs) at the apex. Targeting GSCs remains a challenging task because of their unique biology, resemblance to normal neural stem/progenitor cells, and resistance to standard cytotoxic therapy. Here, we find that the chromatin regulator, JmjC domain histone H3K36me2/me1 demethylase KDM2B, is highly expressed in glioblastoma surgical specimens compared to normal brain. Targeting KDM2B function genetically or pharmacologically impaired the survival of patient‐derived primary glioblastoma cells through the induction of DNA damage and apoptosis, sensitizing them to chemotherapy. KDM2B loss decreased the GSC pool, which was potentiated by coadministration of chemotherapy. Collectively, our results demonstrate KDM2B is crucial for glioblastoma maintenance, with inhibition causing loss of GSC survival, genomic stability, and chemoresistance.

**Abbreviations**
CCNU: lomustine
CI: combination index
GBM: glioblastoma
GSC: glioblastoma stem‐like cell
KDM: lysine‐specific histone demethylase
VP‐16: etoposide

## 1 Introduction

Glioblastoma (GBM) is the most aggressive primary central nervous system (CNS) tumor and is particularly known for its heterogeneity, robust vascularization, and rampant genomic instability. Despite recent advances in standard of care, the median survival of glioblastoma patients in clinical trial remains at approximately 12–15 months (Black *et al*., 2012; Gil *et al*., 2005; Stupp *et al*., 2005). Therapeutic resistance and high recurrence rates in GBM have been attributed to a rare population of cancer stem‐like cells (CSCs) (GBM‐derived CSCs—GSCs), which can be prospectively isolated using a number of molecular markers with prominin‐1 (CD133) being among the most commonly used in current practice (Bao *et al*., 2006; Rich, 2016; Singh *et al*., 2004).

The genome is under constant assault by endogenous factors—such as reactive oxygen species, metabolic intermediates, replication errors; and exogenous factors, such as ultraviolet (UV) radiation and environmental toxins (Bartek *et al*., 2007; Bartkova *et al*., 2005). If left unrepaired, DNA double‐strand breaks (DSBs), caused by these insults, can eventually lead to malignant transformation. DSB repair is orchestrated within the complex organization of chromatin, where chromatin structure and nucleosome assembly represent a significant barrier to the efficient recognition and repair of DSBs (Price and D'Andrea, 2013). Thus, chromatin remodeling and DNA repair are intimately linked and their functional interplay is reflected in the genomic stability of cells. The contribution of epigenetic changes to GBM biology has been studied mostly in terms of aberrant promoter methylation‐induced gene silencing (Maleszewska and Kaminska, 2013); however, emerging evidence points to the role of chromatin remodeling in genome stability maintenance, thereby raising considerable interest in chromatin‐modifying enzymes for targeted therapies (Mack *et al*., 2016).

Histone methylation occurs naturally throughout the genome, mostly at the CpG islands, a subset of which is bound by polycomb‐group proteins, including polycomb repressive complex 1 (PRC1). KDM2B, a Jumonji (JmjC) domain histone histone 3 lysine 36 (H3K36) di‐demethylase, is highly expressed in embryonic stem cells, hematopoietic stem cells, leukemia, and many solid cancers (Farcas *et al*., 2012; He *et al*., 2008, 2011, 2013; Inagaki *et al*., 2015; Karoopongse *et al*., 2014; Tzatsos *et al*., 2009, 2013). In addition to its role in H3K36 and H3K4me3 demethylation, KDM2B recruits PRC1 to CpG islands (Farcas *et al*., 2012), thereby repressing the expression of genes regulating senescence, apoptosis, ribosomal RNA expression, hematopoietic stem cell self‐renewal, and the proper generation of the neural tube *in vivo* (Andricovich *et al*., 2016; Liang *et al*., 2012; Tzatsos *et al*., 2009, 2013).

In this study, we demonstrate that KDM2B plays an important role in GBM cell survival, DNA repair, and maintenance of GSC pools. Importantly, the impact of KDM2B loss or inhibition on the survival and DNA repair capacity of GBM cells is further potentiated when combined with either lomustine (CCNU) or etoposide (VP‐16), chemotherapeutics routinely used in therapeutic management of recurrent disease (Taal *et al*., 2014; Wick *et al*., 2017).

## 2 Materials and methods

### 2.1 Primary cell cultures and glioma patient tissue

About 4121 and 4302 cells were a generous gift from J.N. Rich (University of California, San Diego, CA, USA). IN84 cells were a generous gift from I. Nakano (University of Alabama, Birmingham, Alabama, USA). Primary glioblastoma cell cultures (T115, 1587, T131, T133, T140, T143, T91, and 017) were established upon the approval by Danish Ethical Committee/Danish Data Protection Agency (2006‐41‐6979/KF‐01‐327718). Signed informed consents were obtained 24 h prior surgery for each patient. Primary patient‐derived glioblastoma cell cultures were cultured in stem cell‐permissive Neurobasal®‐A media (NB) (Invitrogen, Taastrup, Denmark; #10888‐022) supplemented with B27 (#12587‐010), bFGF (10 ng·mL^−1^, #13256‐029), EGF (10 ng·mL^−1^, #PHG0311), Glutamax, penicillin (50 U·mL^−1^), and streptomycin (50 μg·mL^−1^, #15070‐063) (all from Invitrogen) with 5% CO_2_ at 37 °C. Glioblastoma patient tissue samples used for protein lysate preparation were collected at surgery and stored in liquid nitrogen before use. Normal human astrocytes (#CC‐2565) were obtained from Lonza and grown in astrocyte growth medium (AGM) with provided supplements. Prior each experiment, cells were dissociated using Accutase (Thermo‐Fisher, Hvidovre, Denmark, #00‐4555‐56) and counted using a NucleoCounter^®^ NC‐200 (ChemoMetec, Lillerød, Denmark). Cells were seeded in appropriate media and treated with lomustine (CCNU, #L5918), GSK‐J4 (#SML0701) from Sigma‐Aldrich (St. Louis, MO, USA), etoposide (VP‐16; 20 mg·mL^−1^, Meda, Denmark) or a vehicle control (DMSO).

### 2.2 siRNA transfection

For siRNA transfection experiments, constructs targeting KDM2B (KDM2B‐1 and KDM2B‐2; #1299001) and siCTRL (si‐negative control duplex, #462001) were obtained from Thermo Fisher Scientific. Cells were transfected with 50 nm of siKDM2B or siCTRL using Lipofectamine^®^ RNAiMAX (#1377150) in Opti‐MEM reduced serum medium (Thermo Fischer Scientific).

### 2.3 Immunoblot analysis

Cells were lysed in whole‐cell lysis buffer (50 mm Tris/HCl, 10% glycerol, 2% SDS) or modified RIPA buffer (50 mm Tris/HCl, 1% NP‐40, 0.25% Na‐deoxycholate, 150 mm NaCl, 1 mm EDTA) supplemented with protease and phosphatase inhibitors, and protein concentrations were estimated by BCA protein assay (Pierce Biotechnology, Rockford, IL, USA). Protein samples were separated on 4–12% NuPAGE Bis/Tris gels (NP0336BOX) (Invitrogen) and electroblotted onto nitrocellulose membranes (Invitrogen, LC2000). The membranes were blocked for 1 h at room temperature (RT) and incubated with primary antibodies in 5% nonfat milk overnight (ON) at 4 °C followed by horseradish peroxidase (HRP)‐conjugated secondary antibodies for 1 h at RT. Blots were developed using either the SuperSignal West Dura Extended Duration Substrate (#34075) or the SuperSignal West Femto Maximum Sensitivity Substrate (#34095) from Thermo Fisher and developed with the Biospectrum Imaging System (UVP, Upland, CA, USA). Primary antibodies used are shown in Table S1.

### 2.4 Viability assays

**Figure 1:**
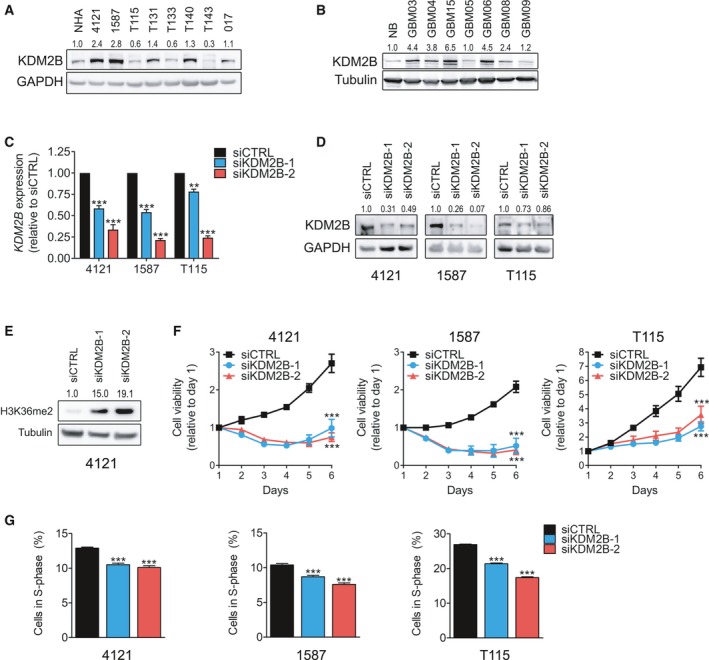
siRNA‐mediated knockdown of KDM2B impairs glioblastoma cell viability and proliferation. (A) Immunoblot analysis of KDM2B and GAPDH (loading control) in glioblastoma cells compared to normal human astrocytes (NHA). (B) Immunoblot analysis of KDM2B and tubulin (loading control) in tissue extracts from glioblastoma samples compared to normal brain (NB). (C) qRT/PCR analysis of relative KDM2B mRNA expression in glioblastoma cells 48 h post‐transfection with either siCTRL or two independent siRNA constructs targeting KDM2B (siKDM2B‐1 and siKDM2B‐2). Data are presented as mean ± SEM,* n* = 4. **P < 0.01; ***P < 0.001 by an unpaired Student's *t*‐test. (D) Immunoblot analysis of KDM2B and GAPDH (loading control) in tumor cells 72 h post‐transfection with siCTRL, siKDM2B‐1, or siKDM2B‐2. (E) Immunoblot analysis of dimethylation of histone H3 at lysine 36 (H3K36me2) and tubulin (loading control) in 4121 glioblastoma cells 48 h post‐siRNA transfection. (F) Relative cell viability of glioblastoma cells over time transfected with siCTRL, siKDM2B‐1, or siKDM2B‐2 constructs analyzed by MTT assay. Data presented as mean ± SEM,* n* = 3. ***P < 0.001 by two‐way ANOVA. (G) ScanR microscopy‐ and software (Olympus)‐based quantification of S‐phase (%, proliferative) cells for 4121, 1587 and T115 glioblastoma lines upon transfection with either siCTRL, KDM2B‐1, or KDM2B‐2 constructs. Data presented as mean ± SD, ***P < 0.001 by one‐way ANOVA followed by Dunnett's *post hoc* test.

**Figure 2:**
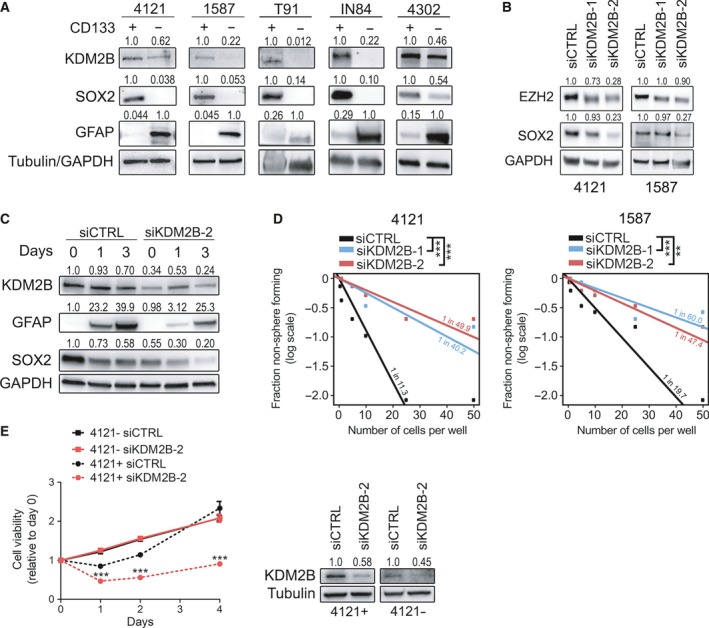
KDM2B is preferentially expressed by GBM‐derived cancer stem‐like cells (GSCs) and is crucial for their maintenance. (A) Immunoblot analysis of KDM2B, SOX2, and GFAP expression in acutely dissociated and MACS‐sorted GSCs and non‐GSCs. Tubulin (4121, 1587, T91) or GAPDH (IN84, 4302) was used as loading control. (B) Immunoblot analysis of EZH2, SOX2, and GAPDH (loading control) in GBM cells 72 h post‐transfection with siCTRL, siKDM2B‐1, or siKDM2B‐2. (C) Immunoblot analysis of KDM2B, GFAP, SOX2, and GAPDH (loading control) in GBM cells that were transfected with either siCTRL or siKDM2B‐2 and subjected to a differentiation assay (exposure to 10% fetal bovine serum) over a period of 3 days. (D) Glioblastoma self‐renewal was analyzed by *in vitro* extreme limiting dilution assay (ELDA). **P < 0.01; ***P < 0.001 analyzed by pairwise chi‐squared test. (E) Cell viability of GSCs (4121+) versus non‐GSCs (4121‐) upon transfection with siCTRL or siKDM2B‐2 and immunoblot analysis of KDM2B knockdown efficiency. Tubulin served as loading control.

The MTT assay (Sigma M‐5655) was employed (Figs 1F, 4, 5A and 6) as previously described (Staberg *et al*., 2017). Cells were plated at a density of 1 × 10^4^ cells per well in 96‐well plates, transfected with siRNA constructs, or treated with GSK‐J4, lomustine, etoposide, or DMSO. After 72 h of incubation, MTT solution was administered and incubated for 4 h followed by addition of 100 μL solubilization buffer. The following day, absorbance was measured at 570 nm with 690 nm as a background reference using a Synergy2 microplate reader with gen5, Microplate Data Collection and Analysis Software (Biotek, Winooski, VT, USA). CellTiter‐Glo Luminescent Cell Viability Assay (Promega, Madison, WI, USA ) was used in Figs 2E, and 5E), where viability was calculated as relative fold change in ATP production with each group internally normalized to the respective vehicle control.

### 2.5 Quantitative real‐time PCR

Total RNA was purified from GBM patient tissue and GBM cell pellets using the QIAshredder (79654) and RNeasy Mini kit (#74104; Qiagen, Copenhagen, Denmark). Synthesis of cDNA and qRT/PCRs was performed using the SuperscriptTM III Platinum^®^ Two Step qRT‐PCR kit with SYBR^®^ Green (Invitrogen, #11735‐032). Gene expression levels were determined applying the comparative Ct method and normalized to expression of three housekeeping genes (TOP1, EIF4A2, and CYC1) included in the human geNorm housekeeping gene selection kit (Primerdesign). Primers used for estimation of mRNA levels were as follows: KDM2B forward; 5′‐CAT GGA GTG CTC CAT CTG CAA TG‐3′, KDM2B reverse; 5′‐ACT TCG GAC ACT CCC AGC AGT T‐3′. Sox2 forward; 5′‐GGC AGC TAC AGC ATG ATG C‐3′, Sox2 reverse; 5′‐TCG GAC TTG ACC ACC GAA C‐3′. Primers were obtained from DNA Technology A/S.

### 2.6 Immunofluorescence imaging

For γH2AX staining and quantification, GBM cells were plated on precoated (Geltrex; Thermo Fisher Scientific) coverslips and either transfected with siRNA constructs, or treated with GSK‐J4 or vehicle control. After 48 h, cells were fixed and stained for anti‐γH2AX Ser139 (Millipore, Copenhagen, Denmark, #05‐636). Nuclei of the cells were counterstained with DAPI, and pictures were acquired on a Zeiss LSM 700 confocal microscope (Birkerød, Denmark). For quantification, 100 nonoverlapping images were acquired for each condition and scored for > 5 foci per cell using the ScanR screening station (Olympus, Ballerup, Denmark). At least 1000 cells were scored and processed using the scanr analysis software (Olympus).

### 2.7 S‐phase cell quantification using ScanR microscopy analysis

Glioblastoma cells were plated on precoated (Geltrex; Thermo Fisher Scientific) coverslips, transfected with respective siRNA and incubated for 72 h. Prior fixation, cells were pulse‐labeled with 10 μm (5‐ethyl‐2′‐deoxyuridine; EdU) for 20 min and then processed using Click‐iT EdU Alexa Fluor 647 Flow Cytometry Assay Kit (Invitrogen) following manufacturer's instructions. Nuclei of the cells were counterstained with DAPI. For S‐phase quantification, 100 nonoverlapping images were acquired for each condition and at least 1000 cells were scored and processed using the scanr analysis software (Olympus). To quantify the percentage of S‐phase cells, single cells were gated based on DAPI—area signal intensity, circularity (to exclude cell debris and doublets), and EdU positivity.

### 2.8 Magnetic‐activated cell sorting

Enrichment of CD133‐positive GBM cells was accomplished using magnetic‐activated cell sorting as per the manufacturer's recommendations (MACS; Miltenyi Biotec, Bergisch Gladbach, Germany). CD133‐positive cells were maintained as neurospheres in stem cell‐permissive Neurobasal^®^‐A media (NB) (Invitrogen, #10888‐022) supplemented with B27 (#12587‐010), bFGF (10 ng·mL^−1^, #13256‐029), EGF (10 ng·mL^−1^, #PHG0311), Glutamax, penicillin (50 U·mL^−1^), and streptomycin (50 μg·mL^−1^, #15070‐063) (all from Invitrogen) with 5% CO_2_ at 37 °C., whereas CD133‐negative cells (non‐GSCs) were maintained in DMEM supplemented with 10% FBS, penicillin (50 U·mL^−1^), and streptomycin (50 μg·mL^−1^, #15070‐063) (all from Invitrogen).

### 2.9 Extreme limiting dilution assay

Glioblastoma cells were dissociated using Accutase, counted, and plated in 96‐well plates at cell densities ranging from 1 to 50 cells/well (16 replicate wells per condition). Transfected cells or cells treated with GSK‐J4, CCNU, or VP‐16 were incubated at 37 °C for 10 days. The formation of tumor spheres was evaluated after 10 days, and each well was analyzed for presence or absence of at least one tumor sphere. The calculation of estimated stem cell frequency in each condition was made by employing the extreme limiting dilution analysis (Hu and Smyth, 2009).

### 2.10 Combination Index calculations

To assess the efficacy of combinational treatments on cell viability (MTT), the free available compusyn software for calculation of a combination index (CI) was used (Chou, 2010). From this, a CI > 1.1 indicates antagonism, a CI of 0.9‐1.1 indicates additivity, and a CI < 0.9 indicates synergy. The CI values calculated were obtained from at least three independent experiments and presented as mean ± standard error of the mean.

### 2.11 Statistics

Data in figures are presented as mean ± standard deviation (SD) or standard error of mean (SEM). All statistical analysis and creation of figures were performed using graphpad prism (v. 7.02, GraphPad, San Diego, CA, USA).

## 3 Results

### 3.1 KDM2B is required for glioblastoma cell survival

Based on the overexpression and oncogenic function of KDM2B in systemic cancers that contain cancer stem cells (Kottakis *et al*., 2014; Tzatsos *et al*., 2013; Ueda *et al*., 2015), we investigated KDM2B as a molecular target in glioblastoma. First, we analyzed its expression in a cohort of primary glioblastoma cell cultures and tissue extracts. qRT/PCR and immunoblot analysis confirmed the expression of KDM2B (Figs 1A and S1A). KDM2B protein levels were elevated in five of eight primary cell cultures compared to nonmalignant control normal human astrocyte cells (NHA). To exclude the possibility that elevated KDM2B expression was a cell culture artifact, we performed qRT/PCR and immunoblot analysis on total RNA and protein isolated directly from primary tumor tissue biopsies. KDM2B expression varied among the individual GBM patients, but was overall higher than that of normal brain control(s) (Figs 1B and S1B,C).

To interrogate the functional role of KDM2B in glioblastoma biology, we selected three representative primary cultures: KDM2B—high‐expressing cells (4121, 1587) and KDM2B—low‐expressing cells (T115). Successful siRNA‐mediated knockdown of KDM2B was confirmed by qRT/PCR and immunoblot analysis (Fig. 1C,D). In addition, KDM2B knockdown was functionally by validated by an increase in the methylation mark inhibited by KDM2B, histone 3 lysine 36 dimethyl, H3K36me2 (Fig. 1E). KDM2B loss impaired glioblastoma cell viability, with greater impact in cells that express higher baseline levels of KDM2B (Fig. 1F). Moreover, KDM2B depletion reduced the fraction of actively proliferating cells (S‐phase) (Fig. 1G).

### 3.2 Targeting KDM2B ablates glioblastoma cancer stem‐like cells

Widespread epigenetic reprogramming occurs during both stem cell differentiation and malignant transformation (Amente *et al*., 2013). Recent findings indicate that chromatin dysregulation is likely to play a crucial role in GBM and the dependence of GSCs on epigenetic regulators offers an opportunity to target their self‐renewal capacity (Jin *et al*., 2017; Miller *et al*., 2017). Based on prior reports that GSCs are the most aggressive cellular population responsible for glioblastoma recurrence (Bao *et al*., 2006; Rich, 2016) and the role of numerous KDMs in stem cell maintenance (Amente *et al*., 2013; Andricovich *et al*., 2016; He *et al*., 2011, 2013), we hypothesized specific function of KDM2B in GSCs. Prospective enrichment of GSCs through the cell surface marker, CD133, revealed preferential expression of KDM2B in GSCs compared to non‐GSCs (Fig. 2A). To link KDM2B to stem cell regulatory pathways, we interrogated core stem cell regulatory pathways that have been linked to GSC maintenance. Targeting KDM2B decreased both the stem cell transcription factor, SOX2, and the chromatin regulatory enzyme, EZH2 (Fig. 2B). Furthermore, the loss of KDM2B led to impaired differentiation capacity of GSCs (Fig. 2C), their self‐renewal (Fig. 2D), and viability (Fig. 2E). Validation in a broader cohort of patients was supported by an *in silico* analysis of the REMBRANDT glioma dataset, which showed a strong positive correlation between *KDM2B* expression with both *CD133* and *SOX2* (Fig. S2A). Collectively, these data, together with the observed reduction in GSC frequency after KDM2B knockdown, are consistent with a crucial role of KDM2B in GSC maintenance.

### 3.3 KDM2B loss induces DNA damage and apoptosis, and sensitizes glioblastoma cells to chemotherapy

**Figure 3:**
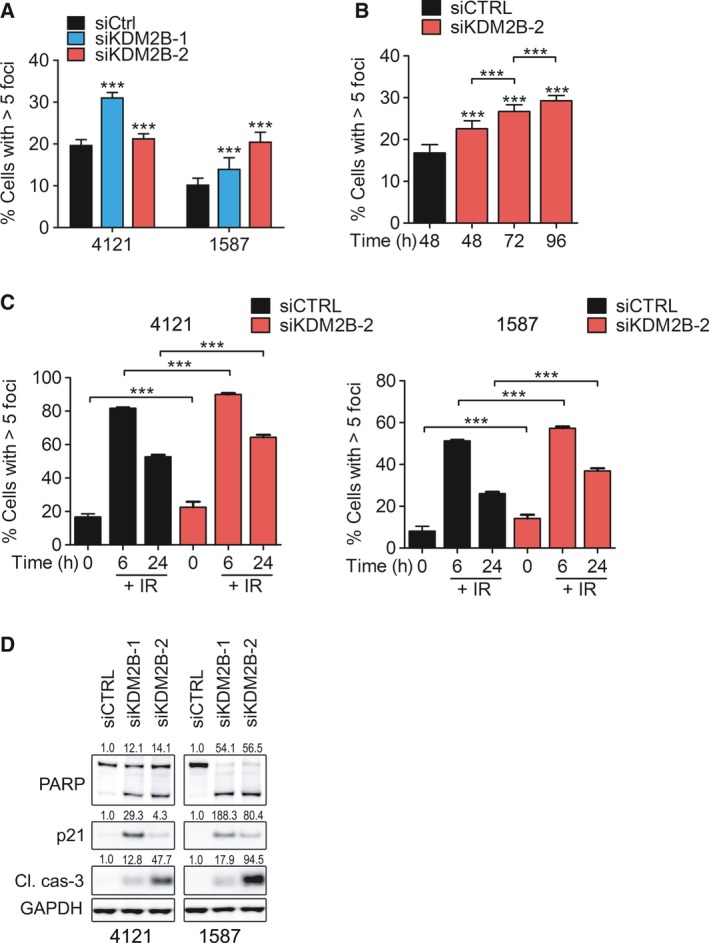
KDM2B loss induces DNA damage, impairs DNA repair capacity of GBM cells, and leads to apoptosis. (A) Quantification of γH2AX Ser139 foci count (% of cells with > 5 γH2AX foci per cell; >1000 cells were analyzed) in 4121 and 1587 cells 72 h post‐transfection with siCTRL, siKDM2B‐1, or siKDM2B‐2. (B) Quantification of γH2AX Ser139 foci count (% of cells with > 5 γH2AX foci per cell; >1000 cells were analyzed) in 4121 cells at 48, 72, and 96 h after transfection with siCTRL, siKDM2B‐1, or siKDM2B‐2. (C) 4121 cells were transfected with either siCTRL or siKDM2B‐2, irradiated (IR, 3 Gy) or sham‐irradiated, and the quantification of γH2AX Ser139 foci count (% of cells with > 5 γH2AX foci per cell; >1000 cells were analyzed) was performed at 0, 6, and 24 h after IR. ***, *P* < 0.001 analyzed by one‐way ANOVA followed by Dunnett's *post hoc* test. (D) Immunoblot analysis of cleaved/total PARP, p21CIP1/WAF1, cleaved caspase‐3, and GAPDH (loading control) in glioblastoma cells 72 h post‐transfection with siCTRL, siKDM2B‐1, or siKDM2B‐2.

Open chromatin augments sensitivity to DNA damage, and the KDM2 family regulates DNA damage repair and correlates with treatment resistance in several cancer types (Banelli *et al*., 2015; Ramadoss *et al*., 2017). To interrogate KDM2B in the genomic stability and DNA repair capacity of glioblastoma, we targeted KDM2B with two nonoverlapping siRNA species (siKDM2B‐1 and siKDM2B‐2). KDM2B loss resulted in a continuous increase in γ‐histone 2AX (γH2AX) foci count over a period of 96 h, an indication of DNA damage accumulation due to impaired DNA repair (Fig. 3A,B). Next, we exposed GBM cells transfected with siCTRL or siKDM2B‐2 to ionizing radiation (IR, 3 Gy) and assessed their DNA repair capacity at 6 and 24 h post‐IR. As shown in Fig. 3C, in addition to baseline increase in γ‐histone 2AX (γH2AX) foci count, the loss of KDM2B impaired the ability of these cells to repair IR‐induced DNA damage. Concordant with reduced proliferation and prevalence of double‐stranded DNA breaks (DSBs), KDM2B knockdown induced p21^CIP1/WAF1^ levels and increased apoptosis, measured by elevated cleavage of PARP and caspase‐3 (Fig. 3D).

**Figure 4:**
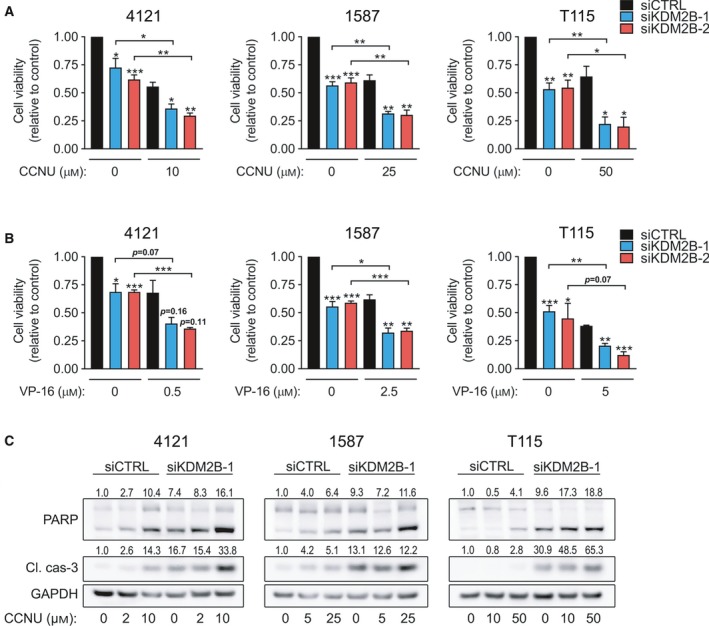
siRNA‐mediated knockdown of KDM2B sensitizes glioblastoma cells to CCNU and VP‐16 chemotherapy. (A, B) Glioblastoma cells were transfected with siCTRL, siKDM2B‐1, or siKDM2B‐2 and treated with either (A) CCNU or (B) VP‐16 at indicated concentrations. Viability was measured 72 h later by MTT assay. Viability measurements are normalized to siCTRL untreated (0 μm) cells (mean ± SEM,* n* = 3). *P < 0.05; **P < 0.01; ***P < 0.001 analyzed by an unpaired *t*‐test. (C) Immunoblot analysis of cleaved/total PARP, cleaved caspase‐3, and GAPDH (loading control) in glioblastoma cells transfected with either siCTRL or siKDM2B‐1 constructs followed by treatment with indicated concentrations of CCNU for additional 48 h.

The heterogeneity of GBM suggests that combination regimens exerting antitumor effects through different targets may be successful in increasing antitumor efficacy (Qazi *et al*., 2017). No standard of care is established in recurrent or progressive GBM. As topoisomerases are involved in DNA repair mechanisms, various combinations with DNA alkylating agents have been tested. This group of antineoplastic agents is cell cycle‐dependent and cycle phase‐specific, and includes irinotecan, topotecan, etoposide (VP‐16), and teniposide (Leonard and Wolff, 2013). Good bioavailability and low toxicity make topoisomerase inhibitors promising candidates for investigation in phase I and II trials. Nitrosoureas, such as carmustine (BCNU), lomustine (CCNU), nimustine (ACNU), and fotemustine, are DNA alkylating agents and have been extensively used in glioma treatment. The use of nitrosoureas increased for recurrent disease when TMZ became standard of care in newly diagnosed glioblastoma (van den Bent *et al*., 2017; Wick *et al*., 2017). The combination of lomustine plus bevacizumab showed prolonged median PFS and OS and higher PFS‐6 than the single agents in the BELOB phase II trial (Taal *et al*., 2014; Wick *et al*., 2017). Therefore, we sought to evaluate to combinational effect of KDM2B targeting KDM2B with selected chemotherapeutics, CCNU and VP‐16. As shown in Fig. 4A,B, KDM2B knockdown, in combination with both CCNU and VP‐16, was more effective at reducing cell viability than either of the monotherapies alone. Genetic KDM2B disruption augmented chemotherapy‐induced apoptosis, as measured by cleavage of PARP and caspase‐3, in all primary glioblastoma cultures tested (Fig. 4C). Collectively, our data indicate that KDM2B promotes chemotherapy resistance in GBM.

### 3.4 Pharmacological inhibition of KDM2B targets GSCs

**Figure 5:**
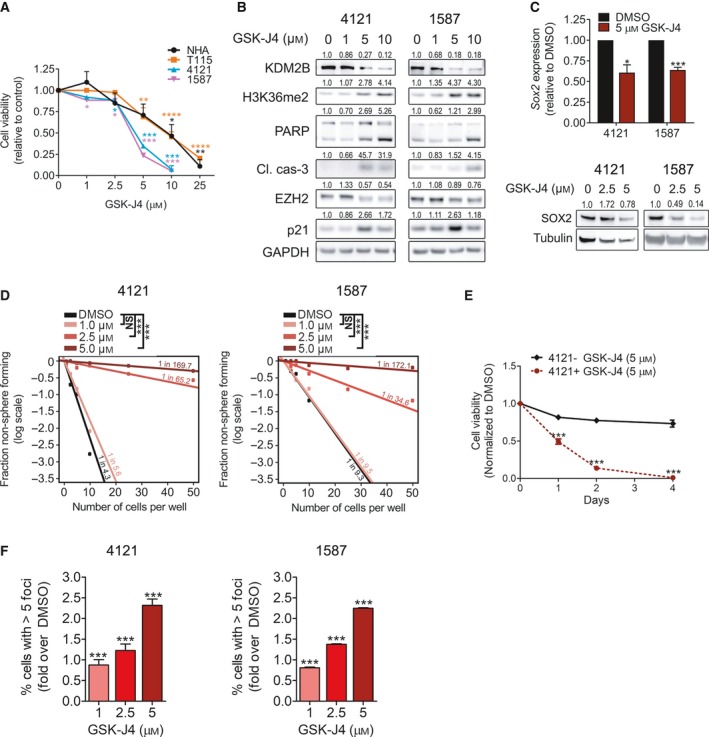
GSK‐J4 treatment impairs glioblastoma cell viability and self‐renewal, and induces apoptosis and DNA damage. (A) Glioblastoma cells and normal human astrocytes (NHA) were treated with increasing concentrations of GSK‐J4, and cell viability was assessed 72 h later by MTT assay. Data are presented as mean ± SEM,* n* ≥ 2. *P < 0.05; **P < 0.01; ***P < 0.001 analyzed by one‐way ANOVA. (B) Glioblastoma cells were treated with increasing concentrations of GSK‐J4 for 48 h and submitted to immunoblot analysis of KDM2B, cleaved/total PARP, EZH2, cleaved caspase‐3, p21CIP1/WAF1, H3K36me2, and GAPDH (loading control) (C) qRT/PCR analysis of relative *SOX2* mRNA expression in glioblastoma cells treated with DMSO or 5 μm GSK‐J4 for 48 h. Data are presented as mean ± SEM,* n* = 3. *P < 0.05; ***P < 0.001 analyzed by an unpaired *t*‐test. Additionally, glioblastoma cells were treated with increasing concentrations of GSK‐J4 for 48 h and subjected to immunoblot analysis of Sox2 and tubulin (loading control). (D) GSC self‐renewal after GSK‐J4 treatment was analyzed by *in vitro* extreme limiting dilution assay (ELDA). ***P < 0.001 analyzed by pairwise chi‐squared test. (E) 4121 GSCs were treated with increasing concentrations of GSK‐J4 for 24 h (as used in ELDA) and the percentage of cells (%) with > 5 γH2AX foci per cell (>1000 cells were analyzed) was quantified. ***P < 0.001 analyzed by one‐way ANOVA followed by Tukey's *post hoc* test.

Based on the efficacy of genetic targeting of KDM2B against glioblastoma, we investigated the efficacy of novel enzymatic inhibitors of KDM2B function. GSK‐J4 is among the first highly potent small‐molecule KDM inhibitors, with efficacy against brainstem gliomas harboring histone 3.3 K27M mutations (Hashizume et al., 2014; Heinemann et al., 2014). While GSK‐J4 was originally described as a specific inhibitor of KDM6A and KDM6B, subsequent analysis reveals that it targets a broader spectrum of the KDM family, including KDM2B (Heinemann *et al*., 2014). GSK‐J4 treatment decreased glioblastoma cell viability in a concentration‐dependent manner, with two cultures expressing high KDM2B levels (4121 and 1587) displaying higher sensitivity compared to glioblastoma cultures expressing low KDM2B levels (T115) and normal astrocytes (Fig. 5A). GSK‐J4 reduced KDM2B expression and increased H3K36me2, the biomarker inhibited by KDM2B, associated with induction of p21^CIP1/WAF1^ expression and the cleavage of PARP and caspase‐3 (Fig. 5B). Supporting a role in targeting GSCs, GSK‐J4 treatment reduced levels of GSC regulators, EZH2 and SOX2 (Fig. 5B,C)**,** decreased the self‐renewal as well as survival of GSCs (Figs 5D,E and S2B). Interestingly, the reduction in GSC frequency was associated with concentration‐dependent induction DSB formation (Fig. 5F).

### 3.5 KDM Inhibitor Sensitizes Glioblastoma to Chemotherapy

**Figure 6:**
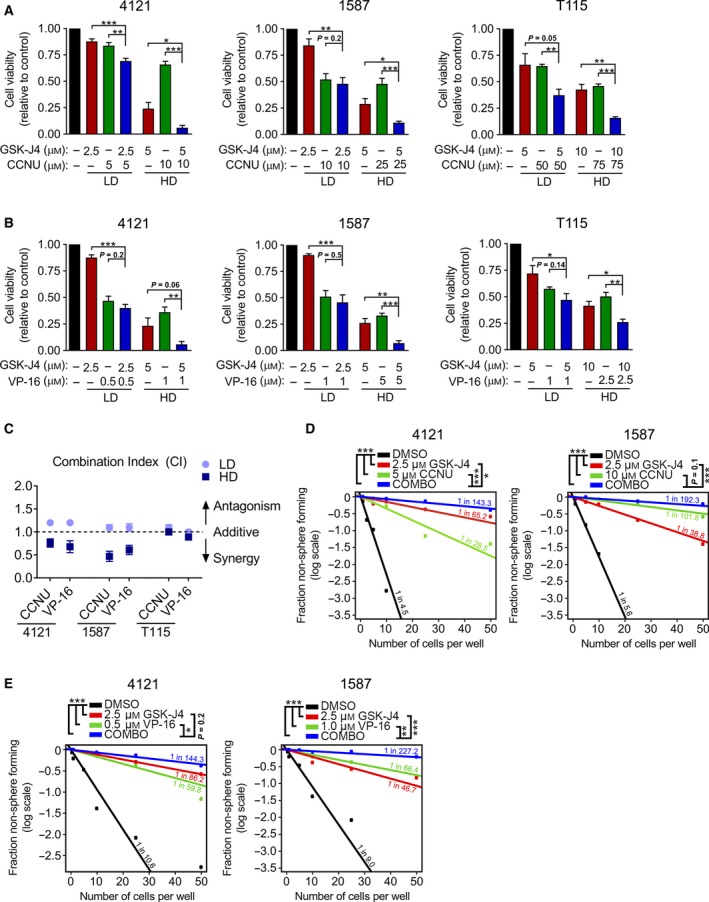
GSK‐J4 treatment sensitizes glioblastoma cells to CCNU and VP‐16 chemotherapy. (A) Glioblastoma cells were treated with low‐dose (LD) or high‐dose (HD) single therapy or a combination of GSK‐J4 and CCNU, and cell viability was assessed by MTT assay. Data presented as mean ± SEM,* n* ≥ 3. *P < 0.05; **P < 0.01; ***P < 0.001 analyzed by an unpaired *t*‐test. (B) Glioblastoma cells were treated with LD or HD single therapy or a combination of GSK‐J4 and VI‐16, and cell viability was assessed by MTT assay. Data presented as mean ± SEM,* n* ≥ 3. *P < 0.05; **P < 0.01; ***P < 0.001 analyzed by an unpaired *t*‐test. (C) Combination index (CI) was calculated using the compusyn software. CI values < 0.9 indicate synergy, 0.9–1.1 additivity, and >1.1 antagonism. Data presented as mean ± SEM,* n* = 3. (D) Glioblastoma self‐renewal after treatment with GSK‐J4 alone or in combination with CCNU was analyzed by *in vitro* extreme limiting dilution assay (ELDA). *P < 0.05; ***P < 0.001 analyzed by a pairwise chi‐squared test. (E) Glioblastoma self‐renewal after treatment with GSK‐J4 alone or in combination with VP‐16 was analyzed by *in vitro* extreme limiting dilution assay (ELDA). *P < 0.05; **P < 0.01; ***P < 0.001 analyzed by a pairwise chi‐squared test.

Based on the effects of genetic targeting of KDM2B on chemotherapy sensitivity, we interrogated the potential of GSK‐J4 to sensitize glioblastoma cells to CCNU and VP‐16, by treating cells with GSK‐J4 or chemotherapy alone or in combination with one another, permitting calculation of the combinational index (CI) (Fig. 6A–C). Here, both low‐dose (LD) and high‐dose (HD) concentrations based on calculated GI_50_ values were tested in all 3 GBM lines. Whereas LD combinations failed to show benefit (synergy or additive), the HD combination of GSK‐J4 with either CCNU or VP‐16 showed significant synergistic inhibition (CIs < 0.9) of cell viability in only KDM2B—high‐expressing 4121 and 1587 cells (Fig. 6A–C). Supporting the functional impact of a combined treatment strategy, treatment with GSK‐J4 and either CCNU or VP‐16 decreased GSC frequencies in both 4121 and 1587 cells (Fig. 6D,E). Collectively, these results support combinatorial benefit of KDM inhibition with herein tested chemotherapeutic agents CCNU and VP‐16 for glioblastoma therapy.

## 4 Discussion

Emerging evidence suggests that epigenetic dysregulation plays fundamental roles in the onset and maintenance of cancer. Numerous studies have shown that standard‐of‐care cancer therapies induce drug‐resistant phenotype that is largely reversible, strongly suggesting benefit from interventions of epigenetic mechanisms (Black *et al*., 2012; Hashizume *et al*., 2014; Hojfeldt *et al*., 2013; Kampranis and Tsichlis, 2009; Kreth *et al*., 2014; Mack *et al*., 2016; Maes *et al*., 2015). Reversible histone methylation has emerged in the last decade as an important element contributing to the development of several diseases, especially cancer (Hoffmann *et al*., 2012). Pathophysiologically, there are strong links between the expression of certain histone demethylases, chromatin remodeling, and the initiation as well as maintenance of cancer (Hashizume *et al*., 2014; Maes *et al*., 2015; Pedersen and Helin, 2010; Rotili and Mai, 2011).

Despite concerted worldwide efforts to tackle glioblastoma, patient prognosis remains grim and recurrence is inevitable (Rich, 2016; Stupp *et al*., 2005). Several histone demethylases, including LSD1, KDM5A, and KDM5B, are upregulated in malignant gliomas, sustaining cell growth, and resistance to chemotherapy (Amente *et al*., 2015; Banelli *et al*., 2015; Black *et al*., 2013; Dai *et al*., 2014; Hayami *et al*., 2011; Tzatsos *et al*., 2013). In the present study, we examined the role of KDM2B in glioblastoma. We found that KDM2B is expressed in glioblastoma and critically maintains glioblastoma cell survival, genome integrity, and stem‐like tumor populations. KDM2B dependency of glioblastoma cells is supported by the preferential sensitivity of KDM2B^high^ tumor cells to KDM2B inhibition compared to KDM2B^low^ tumor cells. RNA interference against KDM2B led to a massive induction of genotoxic stress, cell cycle arrest, and consequent apoptotic cell death, consistent with prior studies in pancreatic and breast cancers (Kottakis *et al*., 2014; Tzatsos *et al*., 2013).

In mammalian cells, double‐strand DNA breaks (DSBs) are repaired by either error‐free homologous recombination (HR) or error‐prone nonhomologous end‐joining (NHEJ) repair pathways (Brandsma and Gent, 2012). H3K36me2 methylation near DSBs is crucial for NHEJ repair after ionizing radiation, and according to the report by Jiang *et al*. (2015), this methylation can be counteracted by the expression of KDM2B. This study found local fumarate to inhibit KDM2B activity, which led to enhanced recruitment of NHEJ repair factors to DSBs, more efficient repair and cell survival (Jiang *et al*., 2015). Kurt and coworkers recently proposed a novel role of KDM2B in mediating the repression of proapoptotic proteins (Kurt *et al*., 2017). In line with these observations, our study finds genetic or pharmacologic KDM2B targeting induced DSBs, cleavage of proapoptotic proteins PARP and caspase‐3.

KDM2B contributes to PRC1 complex recruitment to CpG islands (Farcas *et al*., 2012). BMI1 is a core component of PRC1 and facilitates DSB repair (Ginjala *et al*., 2011). Future studies will determine whether KDM2B loss impairs BMI1 recruitment to DSBs and sites of DNA damage response (DDR) activation, resulting in accumulation of deleterious DNA damage. KDM2B‐mediated recruitment of the suppressive PRC1 complex may silence actively transcribed regions, making these available to DNA repair machinery (Price and D'Andrea, 2013).

KDM2B is also required for maintenance of murine embryonic stem cells and breast cancer‐initiating cells (He *et al*., 2013; Kottakis *et al*., 2014). Here, we find KDM2B to be expressed at higher levels in GSCs compared to their differentiated counterparts. GSC frequency, differentiation capacity, and survival decreased upon either KDM2B knockdown or chemical inhibition using GSK‐J4, supporting its importance in GSC self‐renewal and maintenance. Our data indicate that an early induction of DNA damage (24 h post‐treatment) translates into impaired self‐renewal and maintenance of GSCs (Figs 2 and 3). KDM2B loss was accompanied by the reduction in EZH2 and SOX2 protein levels, with both EZH2 and SOX2 serving as key factors in GSC self‐renewal and maintenance (Suva *et al*., 2009). KDM2B silencing is also reported to increase levels of tumor‐suppressing miRNA *let‐7b*, thereby downregulating EZH2 and reducing the entry into S‐phase and thus cancer growth (Karoopongse *et al*., 2014; Tzatsos *et al*., 2011). We observed induction of p21^CIP1/WAF1^ expression, as well as impaired the entry of glioblastoma cells into S‐phase after KDM2B loss. As several KDMs, including LSD1 and KDM5B, have been reported to regulate p21^CIP1/WAF1^ expression, KDM2B may share control of cell cycle kinetics and induction of senescence (Amente *et al*., 2015; Fasano *et al*., 2007; Wong *et al*., 2012).

A common issue in cancer is the rapid emergence of resistant clones after initial therapy leading to recurrence. Recently, two KDMs, KDM3A in ovarian cancer and KDM5A in glioblastoma, were shown to confer resistance to chemotherapy (Banelli *et al*., 2015; Ramadoss *et al*., 2017). Thus, we speculated that KDM2B may contribute to chemoresistance in glioblastoma. GSK‐J4 was originally thought to be a highly specific inhibitor of the H3K27me3/me2‐demethylases, JMJD3 (KDM6B) and UTX (KDM6A). Hashizume and coworkers reported GSK‐J4 as a promising drug candidate in the treatment of pediatric brainstem glioma harboring an oncogenic K27M mutation in histone H3.3 (Hashizume *et al*., 2014), concluding that there was no efficacy against wild‐type H3K27 glioblastoma. This is in contrast to our findings, which showed high sensitivity of glioblastoma cells to GSK‐J4. In addition, both siRNA‐mediated KDM2B loss as well as chemical inhibition using GSK‐J4 significantly sensitized tumor cells to chemotherapeutics used in clinical management of glioblastoma (Taal *et al*., 2014; Wick *et al*., 2017): lomustine and etoposide. GSCs have been reported to be resistant to several chemotherapies, and GSK‐J4 monotherapy targeted GSC frequency and molecular regulators. This effect was potentiated in combination with both lomustine and etoposide. In agreement to our findings of H3K27 wild‐type tumor cell sensitivity to GSK‐J4, ovarian cancer and non‐small‐cell lung cancer are killed by GSK‐J4, irrespective of H3K27 mutational status (Sakaki *et al*., 2015; Watarai *et al*., 2016). Heinemann *et al*. (2014) showed that GSK‐J4 also inhibits the catalytic activity of the other demethylases (KDM2B, KDM3B, KDM4A, KDM4B, KDM4C, KDM5A, KDM5B, KDM5C, and PHF8) with similar potency. Hence, it cannot be excluded that in addition to KDM2B, the antineoplastic effects reported in our study may be in part mediated through inhibition of other KDMs.

In conclusion, we now report that KDM2B is preferentially expressed therapeutically resistant pool of GSCs, creating a dependency for which inhibition leads to accumulation of DNA damage, which if left unrepaired induces apoptosis and significantly reduces the pool of stem‐like tumor cells. Further, KDM2B inhibition sensitizes this highly resistant tumor to chemotherapy, suggesting a potential clinical paradigm combined with standard therapies.

## Author contributions

MS, SRM, RDR, HP, KEJ, and MV conducted *in vitro* studies. KVS assisted with data analysis. JSR, JB, and HSP contributed to patient material collection and cell line derivation; and PH is responsible for study design, data collection/analysis, and manuscript writing.

## Supporting information


**Table S1.** Overview of primary antibodies used for western blotting (WB).
**Fig. S1.** (A) qRT‐PCR analysis of KDM2B mRNA expression in GBM cell cultures compared to normal human astrocytes (NHA), (mean ± SD, technical replicates = 2, *n* = 1). Bar graph (B) and scatter plot graph (C) showing the results of qRT‐PCR analysis of surgical GBM patient samples normalized and compared to the mean of two normal brain samples (NB) (technical replicates = 2), (*n* = 2 for NB, *n* = 1 for each GBM patient; *P*‐value = 0.12).
**Fig. S2.** (A) *KDM2B* expression is positively correlated to *PROM1* (CD133) and *SOX2*, both markers of stemness in GBM. The analysis was performed using the REMBRANDT data set via GlioVis online tool (http://gliovis.bioinfo.cnio.es/). (B) GSK‐J4 reduces the fraction of CD133‐positive GBM cells *in vitro*. GBM cells (4121 and 1587) were plated and treated with increasing concentrations of GSK‐J4 for 72 h. After incubation, cells were stained with an anti‐CD133‐FITC antibody (Miltenyi Biotec #293C3). Dead cells were excluded using 7‐AAD staining. FACS Verse Cell Sorter (BD Biosciences) was used for acquisition and flowjo software for data analysis. Representative FACS plots from one experiment are shown.
