# Supplementary material for: Targeting glioma stem‐like cell survival and chemoresistance through inhibition of lysine‐specific histone demethylase KDM2B
Source: Mol Oncol. 2018 Feb 12;12(3):406–20. doi: 10.1002/1878-0261.12174 (PMC5830623; doi:10.1002/1878-0261.12174)
Supplement: Supplementary file 1 — Table S1. Overview of primary antibodies used for western blotting (WB). Fig. S1. (A) qRT‐PCR analysis of KDM2B mRNA expression in GBM cell cultures compared to normal human astrocytes (NHA), (mean ± SD, technical replicates = 2, n = 1). Bar graph (B) and scatter plot graph (C) showing the results of qRT‐PCR analysis of surgical GBM patient samples normalized and compared to the mean of two normal brain samples (NB) (technical replicates = 2), (n = 2 for NB, n = 1 for each GBM patient; P‐value = 0.12). Fig. S2. (A) KDM2B expression is positively correlated to PROM1 (CD133) and SOX2, both markers of stemness in GBM. The analysis was performed using the REMBRANDT data set via GlioVis online tool (http://gliovis.bioinfo.cnio.es/). (B) GSK‐J4 reduces the fraction of CD133‐positive GBM cells in vitro. GBM cells (4121 and 1587) were plated and treated with increasing concentrations of GSK‐J4 for 72 h. After incubation, cells were stained with an anti‐CD133‐FITC antibody (Miltenyi Biotec #293C3). Dead cells were excluded using 7‐AAD staining. FACS Verse Cell Sorter (BD Biosciences) was used for acquisition and flowjo software for data analysis. Representative FACS plots from one experiment are shown. [file MOL2-12-406-s001.pdf]

SUPPLEMENTARY MATERIAL

Supplementary Table 1. Overview of primary antibodies used for Western blotting (WB). Antibodies are listed with dilution, manufacturer and catalog number.

Dilution	Antibody	Manufacturer
1:1000	Rabbit anti-cleaved caspase-3	Cell signaling, #9664
1:10000	Rabbit anti-GAPDH	Santa Cruz, #sc-25778
1:1000	Mouse anti-Sox2	Millipore, #MAB4343
1:500	Mouse anti-KDM2B	Novus, #H0084678-M09
1:1000	Rabbit anti-tubulin	Cell signaling, #2125
1:1000	Rabbit anti-PARP	Cell signaling, #9542
1:1000	Mouse anti-EZH2	BD Biosciences, #612666
1:1000	Mouse anti-p21 (Waf1/Cip1)	Cell signaling, #2946
1:1000	Rabbit anti-H3K36me2	Cell signaling, #2901
1:1000	Rabbit anti-γH2AX pSer139	Abcam, #ab11174
1:10000	Rabbit anti-GFAP	DAKO, #Z0334

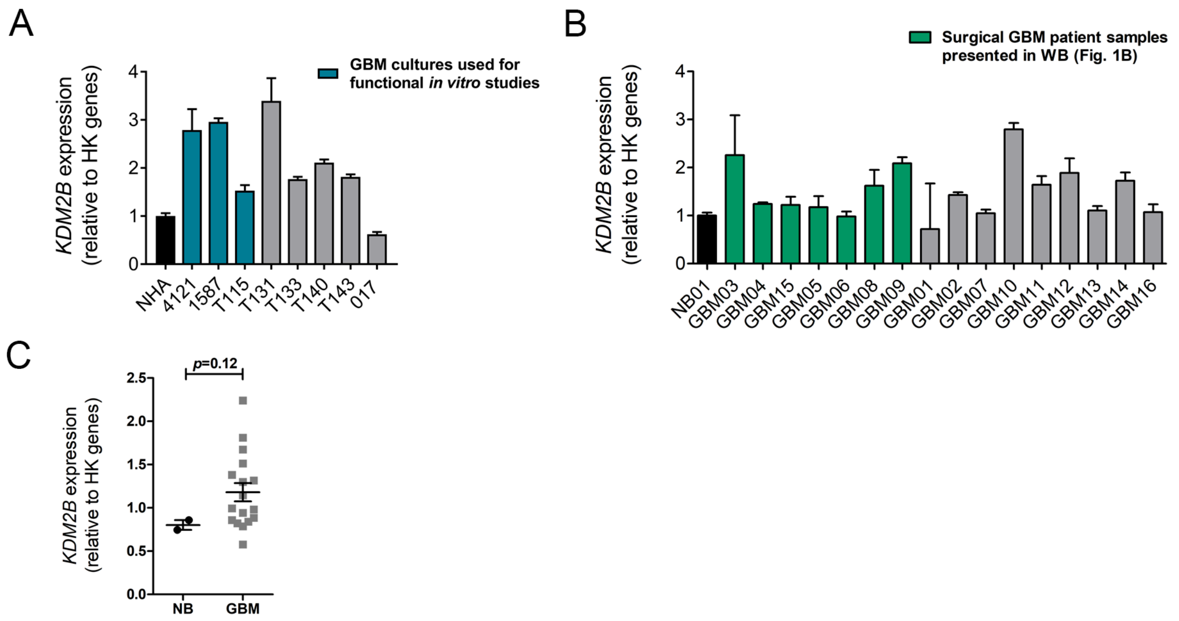

Supplementary Figure S1. (**A**) qRT-PCR analysis of KDM2B mRNA expression in GBM cell cultures compared to normal human astrocytes (NHA), (mean ± SD, technical replicates=2, n=1). Bar graph (**B**) and scatter plot graph (**C**) showing the results of qRT-PCR analysis of surgical GBM patient samples normalized and compared to the mean of two normal brain samples (NB) (technical replicates=2), (n=2 for NB, n=1 for each GBM patient; p-value = 0.12).

A

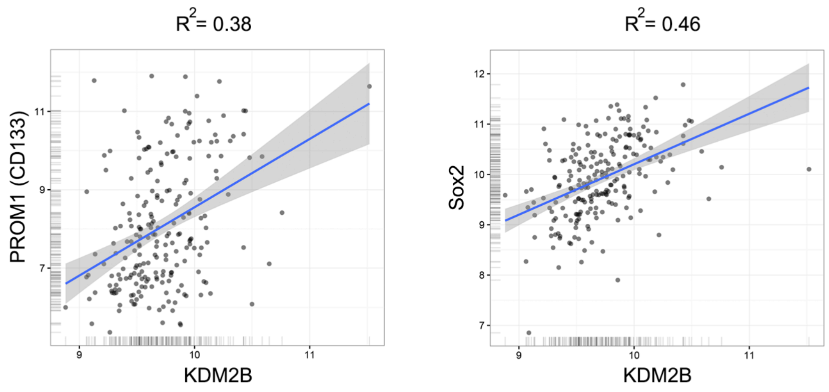

	KDM2B			
	R^2	*p* value	Linear regression analysis	95% confidence interval
**CD133**	0.38	5.7e-09	0.08327	0.26-0.49
**SOX2**	0.45	1.3e-12	0.20684	0.34-0.55

B

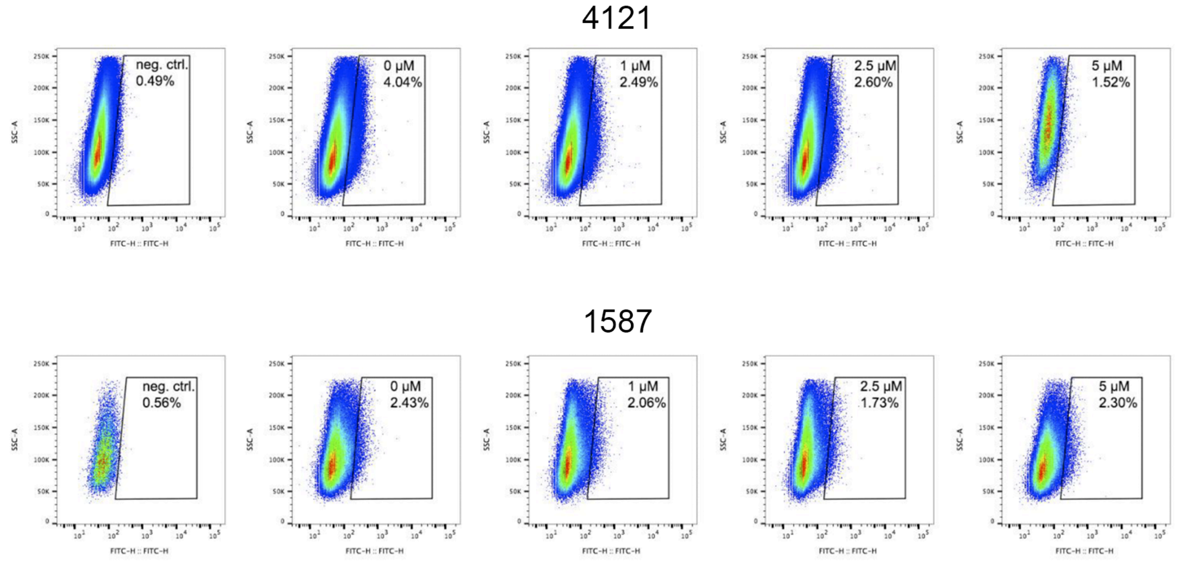

Supplementary Figure S2. (**A**) *KDM2B* expression is positively correlated to *PROM1* (CD133) and *SOX2*, both markers of stemness in GBM. The analysis was performed using the REMBRANDT data set via GlioVis online tool (http://gliovis.bioinfo.cnio.es/). (**B**) GSK-J4 reduces the fraction of CD133-positive GBM cells *in vitro*. GBM cells (4121 and 1587) were plated and

treated with increasing concentrations of GSK-J4 for 72 hours. After incubation, cells were stained with an anti-CD133-FITC antibody (Miltenyi Biotec #293C3). Dead cells were excluded using 7-AAD staining. FACS Verse Cell Sorter (BD Biosciences) was used for acquisition and FlowJo software for data analysis. Representative FACS plots from one experiment are shown.
